# Supplementary material for: Six Steps towards a Spatial Design for Large-Scale Pollinator Surveillance Monitoring
Source: Insects. 2024 Mar 26;15(4):229. doi: 10.3390/insects15040229 (PMC11049859; doi:10.3390/insects15040229)
Supplement: Supplementary file 1 [file insects-15-00229-s001.zip › insects-2871582-SI.pdf]

Six Steps towards a Spatial Design for Large-Scale Pollinator Surveillance Monitoring

Niels Hellwig [1,*], Frank M. J. Sommerlandt [1], Swantje Grabener [1], Lara Lindermann [1], Wiebke Sickel [1], Lasse Krüger [1] and Petra Dieker [1,2]

[1] Thünen Institute of Biodiversity, Bundesallee 65, 38116 Braunschweig, Germany; frank.sommerlandt@thuenen.de (F.M.J.S.); swantje.grabener@thuenen.de (S.G.); lara.lindermann@thuenen.de (L.L.); wiebke.sickel@thuenen.de (W.S.); lasse.krueger@thuenen.de (L.K.); petra.dieker@thuenen.de (P.D.)

[2] National Monitoring Centre for Biodiversity, Federal Agency for Nature Conservation, Alte Messe 6, 04103 Leipzig, Germany

* Correspondence: niels.hellwig@thuenen.de; Tel.: +49-531-596-2579

Supplementary Materials

Conceptual framework for the spatial sampling design

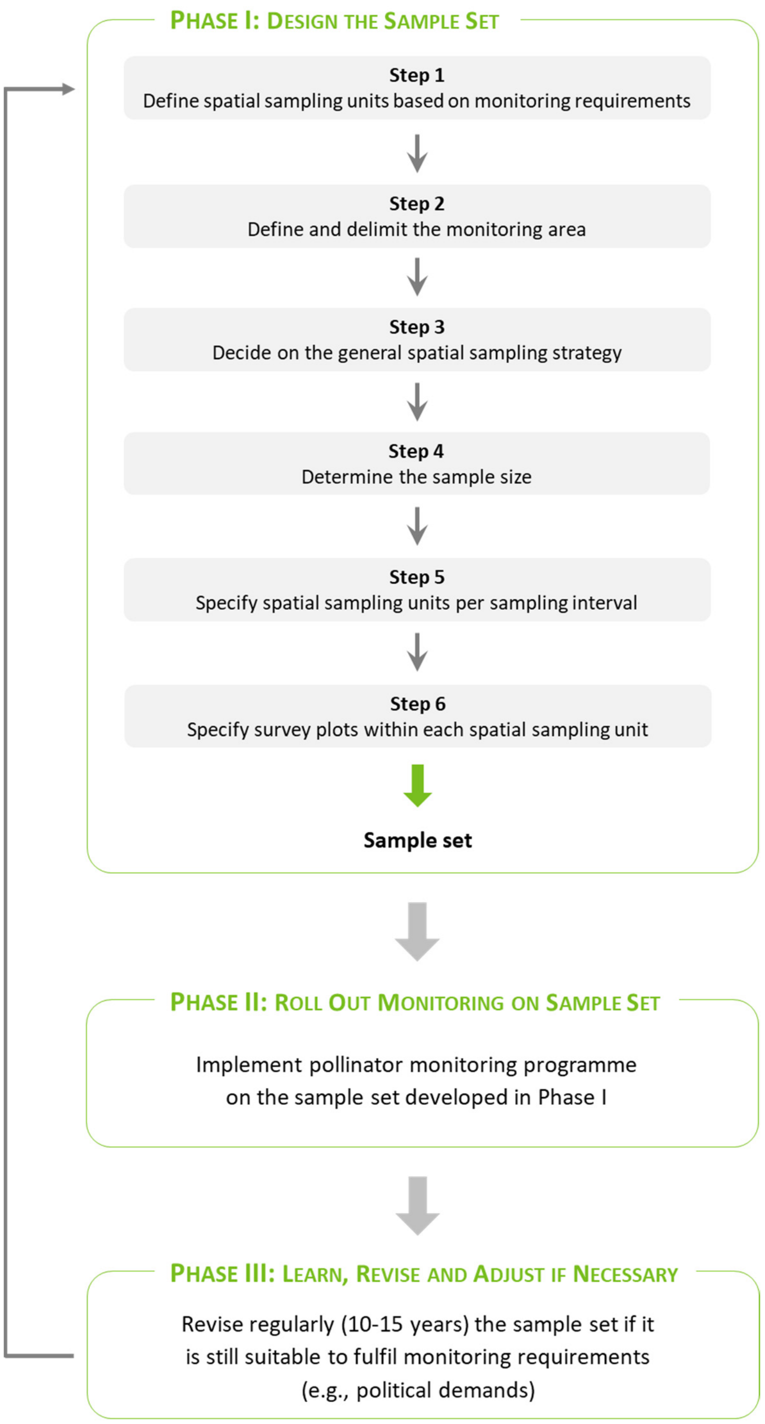

Figure S1. Six steps in spatial sampling design (Figure 2), as embedded and iteratively processed in three phases, which have been proposed in the road map for designing and implementing a monitoring programme by Reynolds et al. [21]. In Phase I, a sample set is designed, Phase II includes the roll-out of the monitoring, and in Phase III, results and experiences gained in the monitoring are used to revise and, if necessary, adjust the sample set. Phase III may be run multiple times due to new insights from the monitoring.

Step 2: Delimit the agricultural landscape

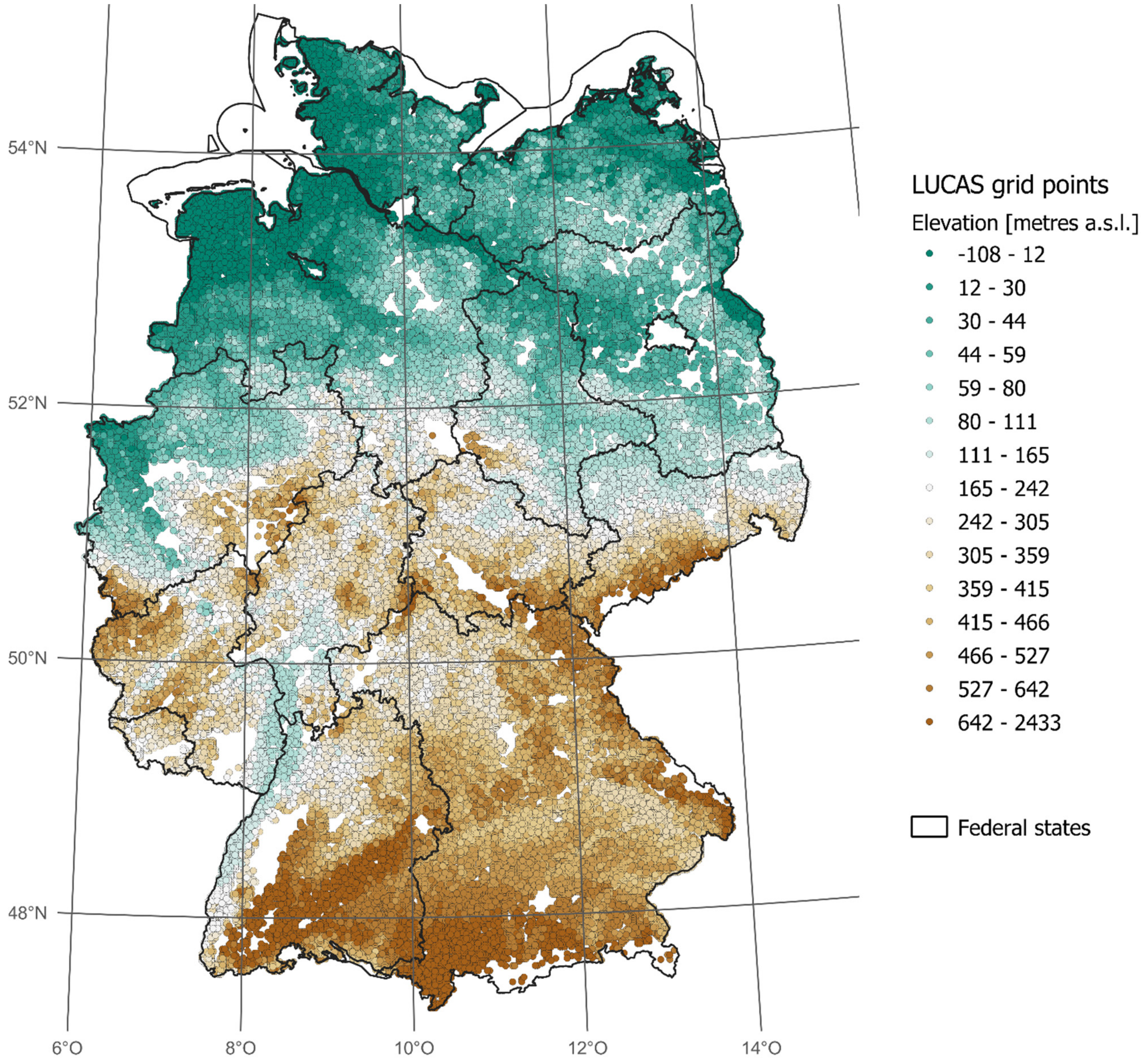

Figure S2. LUCAS grid points preselected by a threshold of 30 % agricultural areas per 3 km x 3 km quadrats and 1 km x 1 km subquadrats.

Step 4: Determine the sample size

Table S1. Species richness of cavity-nesting wild bees in nesting aids as detected by previous studies [101–110].

Species richness	Spatial context	Reference
5	Göttingen, Lower Saxony	Westphal et al. [101]
13	Vaud / Swiss Plateau, Switzerland	Fabian et al. [102]
22	Jena Experiment, Thuringia	Ebeling et al. [103]
10	Wetterau, Hesse	Diekötter et al. [104]
14	Kraichgau, Baden-Württemberg	Gathmann et al. [105]
13	Göttingen, Lower Saxony	Krewenka et al. [106]
5	Schleswig-Holstein	Kruess & Tscharntke [107]
13	Göttingen, Lower Saxony	Steffan-Dewenter [108]
15	Rhenish Hesse, Rhineland-Palatinate	Uzman et al. [109]
14	Swabian Jura, Baden-Württemberg	Steckel et al. [110]
16	Hainich, Thuringia	Steckel et al. [110]
16	Schorfheide, Brandenburg	Steckel et al. [110]

Step 5: Specify locations of landscape quadrats

Table S2. Sample of 950 landscape quadrats for the monitoring of cavity-nesting wild bees in the agricultural landscapes of Germany

LUCAS id	Name	LUCAS id	Name	LUCAS id	Name
40403084	Aachen-Orsbach	41043132	Düsseldorf-Hubbelrath	41522732	Weitenau
40562988	Lahr	41043180	Schermbeck	41522764	Schallstadt-West
40563004	Arzfeld-Ost	41043212	Vitiverter Mark	41522780	Bahlingen-Süd
40563020	Bleialf	41043276	Wilsum-Nordwest	41522796	Rust-Süd
40563052	Alzen	41202956	Niederhambach	41522940	Olsbrücken
40563100	Gereonsweiler	41203020	Düngenheim	41522956	Odenbach
40563116	Gerderath	41203036	Rieden	41523004	Badenhard
40563132	Brüggen-Südost	41203116	Witzhelden	41523020	Brey
40563196	Kleve-Nord	41203148	Essen-Südost	41523036	Vallendar-Nord
40722972	Olk	41203196	Maria Veen	41523052	Dernbach
40723004	Burbach (Eifel)	41203212	Musholt	41523068	Helmenzen
40723020	Gondelsheim	41203228	Heek-West	41523084	Spurkenbach
40723036	Baasem	41203244	Bardel	41523116	Kierspe
40723068	Abenden	41203276	Alte Piccardie	41523132	Hülscheid
40723100	Rödingen	41362732	Huttingen	41523148	Schwerte-Ergste
40723116	Hochneukirch	41362908	Contwig-Ost	41523164	Dortmund-Scharnhorst
40723132	Viersen-Ummer	41362924	Bruchmühlbach-Miesau	41523180	Südkirchen
40723148	Kempen-Nord	41362940	Rammelsbach	41523196	Davert-West
40723164	Issum	41362956	Truppenübungsplatz Baumholder	41523212	Nienberge
40723180	Xanten-Nordwest	41362972	Hennweiler	41523228	Reckenfeld
40723196	Millingen	41362988	Heinzenbach	41523244	Rodde
40882924	Nalbach	41363004	Sabershausen	41523260	Beesten
40882972	Schweich-Ost	41363020	Kalt	41523276	Gersten
40882988	Arenrath	41363036	Andernach-Miesenheim	41523308	Sögel-Nordwest
40883004	Deudesfeld	41363052	Hausen (Wied)	41523324	Börgermoor
40883020	Hohenfels-Essingen	41363068	Buchholz (Westerwald)	41523340	Westoverledingen
40883036	Ahrhütte	41363084	Eischeid	41523356	Veenhusen
40883068	Ülpenich	41363116	Wipperfürth-West	41523372	Ludwigsdorf
40883084	Wissersheim	41363180	Ahsen	41523388	Willmsfeld
40883100	Bergheim-Nordost	41363196	Dülmen-Nordost	41682764	Kirchzarten
40883116	Ramrath	41363212	Billerbeck	41682812	Diersburg
40883132	Meerbusch-Büderich	41363228	Burgsteinfurt-Südwest	41682828	Willstätt-Sand
40883164	Budberg	41363244	Ohne	41682940	Münchweiler an der Alsenz
40883180	Wesel-Nord	41363276	Holthausen-Biene	41683020	Singhofen

41043004	Strotzbüsch	41363308	Oberlangen	41683036	Holler
41043020	Horperath	41363324	Neuheede	41683052	Freilingen
41043052	Freisheim	41363340	Wymeer	41683068	Luckenbach
41043068	Miel	41363372	Loppersum	41683116	Ebberg
41043084	Brühl-Ost	41363388	Wurzeldeich	41683164	Bramey-Lenningsen

41683180	Hamm-Nord	42002972	Dexheim	42322748	Ehingen-Nord
41683196	Sendenhorst-West	42003004	Naurod	42322780	Bubsheim-West
41683212	Telgte-Nord	42003020	Bad Camberg-Südost	42322796	Geislingen-Süd
41683228	Ladbergen-Ost	42003036	Elkerhausen	42322812	Bad Imnau / Starzach
41683244	Ibbenbüren-Ost	42003052	Greifenstein-Allendorf	42322828	Jettingen
41683260	Voltlage	42003100	Erndtebrück-Nordost	42322844	Calw-Ost
41683276	Bippen	42003116	Schmallenberg	42322892	Menzingen
41683292	Löningen-Süd	42003132	Nichtinghausen	42322908	Horrenberg
41683308	Bockholte	42003148	Hirschberg	42322940	Vöckelsbach
41683324	Hilkenbrook	42003164	Altengeseke	42322956	Kolmbach
41683340	Elisabethfehn	42003180	Liesborn	42322972	Roßdorf
41683356	Selverde	42003196	Rheda-Nordwest	42322988	Dietzenbach-Hexenberg
41683388	Negenbargen	42003212	Hesselteich-Süd	42323020	Wöllstadt
41842764	Waldau	42003228	Wellingholzhausen-Südwest	42323036	Rockenberg
41842780	Elz-Hochtal	42003244	Schledehausen	42323068	Hassenhausen
41842860	Rastatt-Wintersdorf	42003260	Vennermoor	42323084	Goßfelden
41842892	Steinweiler	42003276	Steinfeld (Oldenburg)	42323100	Wiesenfeld
41842908	Rhodt unter Rietburg	42003292	Bakum-Süd	42323116	Münden / Neukirchen
41842940	Ebertsheim	42003308	Garther Heide	42323132	Neerdar
41842956	Freimersheim	42003324	Charlottendorf	42323148	Madfeld
41842972	Wörrstadt / Sulzheim	42003356	Lehmden	42323180	Paderborn-West
41842988	Ingelheim-Ost	42162748	Riedöschingen	42323196	Stukenbrock-Senne
41843020	Burgschwalbach	42162764	Oberbaldingen	42323212	Leopoldshöhe
41843036	Limburg-Offheim	42162796	Altoberndorf	42323228	Falkendiek
41843052	Irmtraut	42162812	Haidenhof	42323260	Barl
41843068	Burbach-Lippe	42162972	Griesheim-West	42323276	Dörrieloh / Bleckriede
41843164	Schwefe	42163004	Sulzbach (Taunus)	42323292	Sudbruch
41843180	Stockumer Holz	42163020	Neu-Anspach-Südost	42323308	Dimhausen
41843212	Sassenberg-Nordwest	42163052	Wetzlar-Garbenheim	42323324	Delmenhorst-Südost
41843228	Lienen-Ost	42163084	Silberg	42323356	Wulsbüttel
41843260	Bramsche-Nord	42163100	Beddelhausen	42323372	Düring
41843276	Gehrde-West	42163132	Wiemeringhausen	42323388	Bremerhaven-Nordost
41843292	Osteressen	42163164	Eringerfeld	42323404	Midlumer Heide
41843308	Stalförden	42163180	Mantinghausen	42323420	Duhnen
41843324	Bösel	42163196	Verl-Südwest	42323468	Sankt Peter-Ording
41843340	Dänikhorst	42163228	Spenge-Nordwest	42323532	Hindenburgdamm
41843356	Eggeloge	42163260	Wehdem	42482748	Stockach-Süd
41843372	Horsten	42163276	Rehden-Südwest	42482764	Hölzle

41843388	Jever-Nordost	42163292	Großes Moor / Barnstorf	42482780	Oberglashütte
42002732	Eggingen	42163324	Nuttel	42482812	Ofterdingen-West
42002892	Leimersheim	42163340	Köterende	42482860	Heimerdingen-Ost
42002908	Schwegenheim	42163356	Strückhausermoor	42482876	Hohenhaslach-West
42002924	Dannstadt	42163372	Esenshamm	42482892	Stetten am Heuchelberg
42002940	Beindersheim	42163532	Sylt	42482908	Helmhof
42482940	Beerfelden	42643148	Wettesingen	42803276	Nöpke
42482956	Zell im Odenwald	42643164	Eissen	42803292	Lichtenhorst-Nord
42483020	Lindheim	42643180	Brakel	42803308	Lehringen
42483036	Borsdorf	42643196	Ruensiek	42803324	Kirchwalsede
42483052	Lauter	42643212	Sonneborn	42803340	Scheeßel-Westerholz
42483068	Deckenbach	42643244	Nienstädt	42803356	Weertzen
42483100	Grüsen	42643260	Loccum	42803372	Brest
42483132	Ober-Waroldern	42643276	Estorf	42803388	Hammah-Mittelsdorf
42483148	Kohlgrund	42643292	Schweringerberg	42803404	Krautsand
42483164	Holtheimer Wald	42643308	Eitzendorf	42803420	Hochfeld
42483180	Schwaney	42643324	Steinberg	42803436	Wacken
42483212	Lemgo-Südost	42643340	Dipshorn	42803452	Offenbüttel
42483228	Kalletal-Kalldorf	42643356	Rhadereistedt	42803468	Ekel
42483260	Kreuzkrug	42643372	Bremervörde-Süd	42803484	Hollingstedt
42483276	Eckershausen	42643388	Oste / Eschhornmoor	42803500	Eggebek
42483292	Mellinghausen-Brake	42643404	Oberndorf	42962732	Bodnegg-West
42483308	Wachendorf	42643436	Gudendorf	42962748	Baindt
42483324	Bremen-Südost	42643452	Heide-Südost	42962764	Bad Schussenried-Süd
42483340	Lilienthal	42643468	Kleve / Eider	42962780	Ahlen / Rupertshofen
42483356	Bornreihe	42643484	Mildstedt	42962796	Schlechtenfeld
42483372	Appeln	42643500	Norstedt	42962812	Heroldstatt-Süd
42483388	Lintig	42643516	Hörup	42962860	Rienharz
42483404	Ihlienworth	42802732	Raderach	42962876	Glashofen
42483436	Friedrichskoog	42802748	Hasenweiler	42962924	Assamstadt-West
42483452	Westerdeichstrich	42802780	Altheim-Süd	42962940	Oberlauda-West
42483468	Kating	42802812	Dottingen	42962956	Böttigheim-Nord
42483516	Risum-Lindholm	42802828	Unterlenningen / Erkenbrechtsweiler	42962972	Karbach
42483532	Süderlügum	42802844	Plochingen / Reichenbach	42962988	Halsbach
42642748	Frickingen-Altheim-Nord	42802860	Lehnenberg	42963020	Mottgers
42642764	Ettisweiler	42802924	Osterburken	42963036	Niederkalbach
42642796	Mägerkingen-Süd	42802940	Waldstetten	42963100	Sterkelshausen
42642828	Reutlingen-Mittelstadt	42802956	Sonderriet	42963132	Oberkaufungen
42642844	Stuttgart-Plieningen	42802988	Neuhütten	42963164	Heisebeck
42642860	Stuttgart-Mühlhausen	42803036	Freiensteinau-Ost	42963196	Stadtoldendorf

42642876	Mundelsheim	42803068	Schwarz	42963212	Wallensen
42642908	Höchstberg	42803084	Olberode	42963228	Eldagsen
42642924	Muckental	42803100	Homberg (Efze)-Süd	42963244	Ronnenberg-Weetzen
42642956	Kleinheubach	42803116	Böddiger	42963260	Garbsen-Nordost
42643036	Gedern	42803148	Grebenstein	42963276	Vesbeck
42643052	Ulrichstein	42803164	Trendelburg-Deisel	42963292	Eickeloh
42643068	Romrod-Zell	42803180	Fürstenberg	42963308	Walsrode-Nordost
42643084	Wasenberg	42803196	Wilmeröderberg	42963324	Hiddingen
42643100	Gilsa	42803212	Grohnde	42963340	Benkeloh
42643132	Bründersen	42803260	Wunstorf / Steinhude	42963356	Heidenau
42963372	Apensen	43282924	Finsterlohr	43443260	Ohof
42963404	Elmshorn-Südwest	43282940	Hemmersheim	43443276	Helmerkamp
42963436	Silzen	43282972	Seligenstadt	43443292	Marwede
42963452	Brinjahe	43282988	Rundelshausen	43443324	Allenbostel
42963484	Geltorf	43283004	Oerlenbach-West	43443340	Embsen
42963500	Kattbek	43283020	Steinach	43443356	Bardowick / Handorf
42963516	Husbyholz	43283036	Oberelsbach-West	43443372	Geesthacht-Nordwest
43122716	Siebers	43283052	Feldaquelle (Rhön)	43443404	Lasbek-Gut
43122732	Wangen-Nordost	43283068	Dermbach-West	43443420	Dreggers
43122748	Immenried-Nord	43283100	Breitzbach	43443436	Muggesfelde
43122764	Füramoos	43283132	Dieterode	43443452	Wittmoldt
43122780	Maselheim	43283148	Bischhausen	43443468	Schlesen
43122796	Achstetten-Nord	43283196	Dannhausen	43602732	Görisried
43122812	Herrlingen	43283212	Bönnien	43602748	Huttenwang
43122844	Lauterstein	43283228	Farmsen	43602764	Helchenried
43122892	Unteraspach	43283244	Mehrum	43602780	Zaisertshofen
43122924	Herrenzimmern	43283276	Altencelle	43602796	Muttershofen
43122940	Bernsfelden-Nord	43283356	Tangendorf	43602828	Fristingen-West
43123004	Untererthal	43283372	Hamburg-Ochsenwerder	43602860	Nördlingen-Ost
43123020	Schildeck	43283404	Tangstedt	43602892	Königshofen an der Heide
43123052	Elters	43283420	Barkenholm	43602924	Hainklingen
43123068	Rasdorf-West	43283436	Willingrade	43602940	Dietersheim-Nord
43123100	Solz	43283452	Groß Buchwald	43602988	Knetzgau
43123116	Waldkappel-Nord	43283484	Sprenge	43603004	Hofheim in Unterfranken-Ost
43123148	Dramfeld	43442716	Wagneritz	43603020	Alsleben
43123196	Brunsen	43442732	Kempten (Allgäu)-Süd	43603036	Haina-Nord
43123228	Emmerke	43442748	Schrattenbach	43603052	Dillstädt
43123260	Altwarmbüchen	43442764	Stephansried	43603100	Friedrichswerth-Nord
43123404	Himmelmoor	43442780	Greimeltshofen	43603116	Großengottern
43123420	Lentföhrden	43442796	Oberegg	43603164	Bad Sachsa
43123436	Arpsdorf	43442828	Obermedlingen	43603212	Schladen
43123452	Nortorf	43442844	Fleinheim	43603228	Wolfenbüttel-Ost

43123468	Bredenbek	43442876	Stödtlen	43603276	Wesendorf-Westerholz
43123500	Ketelsby	43442908	Schorndorf	43603292	Hankensbüttel-West
43123516	Koppelheck	43442940	Wüstphül	43603308	Nettelkamp
43282716	Wiedemannsdorf	43442972	Lülsfeld	43603324	Emmendorf
43282748	Bimmlings	43442988	Grettstadt-Nord	43603356	Lüdersburg
43282764	Tannheim	43443036	Mellrichstadt-Ost	43603372	Witzeeze
43282780	Kirchberg an der Iller	43443052	Herpf	43603388	Niendorf/Stecknitz
43282796	Vöhringen	43443100	Eisenach-Stregda	43603404	Düchelsdorf
43282812	Nersingen-West	43443132	Dingelstädt-Süd	43603420	Eckhorst
43282828	Ballendorf-Nord	43443148	Wintzingerode	43603436	Holstendorf
43282892	Crailsheim-Ost	43443212	Upen	43603468	Stöfs
43282908	Brettheim	43443244	Woltorf	43762732	Nachsee
43762748	Tremmelschwang	43922940	Erlangen-Bruck	44083100	Grammetal-Nord
43762764	Ummenhofen	43922972	Strullendorf-Amlingstadt	44083116	Vogelsberg-Nord
43762780	Langerringen-Südwest	43922988	Windischletten	44083132	Oberheldrungen
43762828	Possenried	43923020	Dörfles-Esbach	44083148	Martinsrieth
43762844	Donauwörth-Riedlingen	43923036	Mausendorf	44083164	Wippra
43762876	Heidenheim-Eggenthal	43923084	Kirchheim-Süd	44083180	Ballenstedt
43762892	Laubenzedel	43923116	Werningshausen	44083196	Hedersleben
43762908	Ziegendorf	43923132	Oberbösa	44083212	Oschersleben-Günthersdorf
43762924	Oberreichenbach	43923148	Kelbra-West	44083228	Ovelgünne
43762940	Borbath	43923196	Halberstadt-Süd	44083260	Jeseritz-West
43762956	Lonnerstadt-Nord	43923212	Schlanstedt	44083276	Schwiesau
43762988	Leppelsdorf	43923228	Helmstedt-Reinsdorf	44083308	Riebau-Nord
43763036	Weitersroda	43923244	Weferlingen / Grasleben	44083324	Klautze
43763084	Mühlberg-West	43923260	Oebisfelde-Weferlingen-Niendorf	44083340	Neu Kaliß
43763100	Molschleben	43923276	Neuferchau	44083356	Klein Krams
43763116	Urleben	43923292	Püggen	44083388	Groß Rogahn
43763132	Gundersleben	43923308	Cheine	44083404	Alt Meteln
43763148	Kleinfurra-Hain	43923324	Krummasel	44083420	Alt Jassewitz
43763196	Wernigerode-Reddeber	43923340	Laake	44242732	Loisach / Lainbach
43763212	Osterwieck-Hessen	43923356	Lübtheen	44242796	Günding
43763228	Schöppenstedt-Nordost	43923372	Körchow	44242812	Eglersried
43763244	Ochsendorf	43923388	Woez	44242828	Göbelsbach
43763292	Waddekath	43923404	Holdorf	44242844	Zuchering
43763308	Varbitz	43923420	Mallentin	44242860	Echenzell
43763324	Bankewitz	43923484	Lemkenhafen	44242908	Neumarkt-Rittershof
43763340	Nahrendorf	44082748	Weilheim in	44242924	Oberrieden

			Oberbayern		
43763356	Amholz	44082796	Oberweikertshofen	44242940	Kirchensittenbach
43763388	Hollenbek	44082812	Sielenbach-Nord	44242956	Weidensees
43763404	Schlagsdorf	44082828	Gollingkreut	44242972	Reizendorf
43763452	Marxdorf	44082844	Wagenhofen	44243084	Milda
43763468	Truppenübungsplatz Putlos	44082860	Ochsenfeld-Ost	44243100	Apolda-Oberndorf
43922732	Wildsteig	44082876	Titting-Kaldorf	44243116	Herrengosserstedt
43922780	Petzenhausen	44082892	Tiefenbach	44243132	Wendelstein
43922796	Mering-Sankt Afra	44082940	Eschenau	44243148	Einsdorf
43922812	Derching	44082956	Hundsboden	44243164	Klostermansfeld
43922828	Neukirchen	44082972	Siegritz	44243180	Mehringen
43922844	Staudheim	44082988	Wotzendorf	44243196	Hecklingen-Nordwest
43922892	Stirn	44083004	Strössendorf	44243212	Altenweddingen-West
43922908	Poppenreuth	44083020	Sichelreuth-Süd	44243228	Irxleben
43922924	Oberasbach	44083084	Tannroda	44243260	Letzlingen-Ost
44243276	Lindstedt	44562796	Moosinning-West	44722908	Bubach an der Naab
44243292	Beese	44562812	Rudlfing	44722924	Dürnsricht
44243308	Arendsee-Gestien	44562828	Airischwand	44722972	Krummennaab
44243324	Kapern	44562844	Mitterstetten	44722988	Manzenberg
44243372	Friedrichsmoor	44562860	Abensberg-Nord	44723036	Schönbrunn
44243404	Liessow	44562892	Buxlohe	44723052	Steinsdorf
44243420	Krassow	44562924	Köfering	44723068	Neugernsdorf
44243436	Boiensdorf	44562940	Atzmannsricht	44723084	Kauern
44402732	Lenggries	44562972	Höflas	44723100	Nedissen
44402812	Kranzberg	44563020	Eppenreuth	44723132	Lützen-Nordost
44402828	Geroldshausen in der Hallertau	44563036	Gebersreuth	44723148	Großkugel
44402844	Geisenfeld-Nord	44563052	Langenbuch	44723164	Brehna-West
44402860	Hüttenhausen	44563068	Auma	44723180	Tornau vor der Heide
44402876	Altmühlmünster	44563084	Lindenkreuz	44723196	Dessau-Nordwest
44402892	Wissing-Ost	44563100	Heideland-Königshofen	44723212	Trüben
44402908	Oberwiesenacker	44563116	Gröbitz	44723244	Tucheim / Paplitz
44402924	Schwend	44563148	Hohenweiden	44723260	Jerichow-Roßdorf
44402940	Holnstein	44563164	Nehlitz	44723292	Gülpe
44402956	Degelsdorf	44563180	Pilsenhöhe	44723308	Kümmernitz
44402972	Neuhof	44563196	Kühren	44723324	Bärensprung
44402988	Dressendorf	44563212	Gehrden	44723340	Kemnitz
44403004	Gundlitz	44563228	Möckern	44723356	Schmolde
44403020	Döbra	44563244	Reesen-Ost	44723404	Hoppenrade
44403068	Kolba	44563260	Elbe-Parey	44723420	Recknitz
44403116	Bad Kösen-West	44563276	Tangermünde-Nord	44723436	Göldenitz
44403132	Albersroda	44563292	Gewerbepark Altmark	44723452	Vogtshagen

44403148	Stedten	44563308	Werben-Ost	44882764	Gunzenham
44403164	Beesenstedt	44563324	Viesecke	44882780	Schlicht
44403180	Beesedau	44563356	Porep	44882796	Reibersdorf
44403196	Neugattersleben	44563372	Gischow	44882812	Hinterskirchen
44403228	Biederitz	44563388	Langenhagen	44882828	Adlkofen
44403244	Heinrichsberg-Nord	44563404	Groß Upahl	44882844	Paindlkofen
44403276	Möringen	44563420	Oettelin	44882860	Allkofen
44403292	Polkau	44563436	Ziesendorf-Buchholz	44882876	Roith
44403308	Seehausen-Süd	44722732	Bayrischzell	44882892	Grubberg
44403324	Wittenberge-Ost	44722764	Holzen	44882908	Bruck in der Oberpfalz
44403340	Karstädt-Ost	44722780	Niederaltmannsberg	44882924	Altendorf-Fronhof
44403356	Stresendorf	44722796	Lengdorf	44882940	Großenschwand
44403388	Prestin	44722812	Kirchberg (Oberbayern)	44882956	Ottenrieth
44403404	Sternberg-Nordwest	44722828	Unterlenghart	44882972	Liebenstein
44403420	Hermannshagen	44722844	Rottenburg an der Laaber-Ost	44883020	Landwüst
44403436	Klein Siemen	44722860	Langquaid	44883052	Lengenfeld-Süd
44562732	Rottach-Egern-Ost	44722876	Pentling	44883068	Fraureuth
44562780	Baldham	44722892	Eitlbrunn	44883084	Crimmitschau-Gösau
44883100	Lödla	45043404	Duckow	45363068	Drebach / Hopfgarten
44883148	Leipzig-Seehausen	45043420	Schorrentin	45363116	Döbeln-Ziegra
44883164	Sauseditz	45043436	Wasdow	45363132	Leuben
44883196	Wörlitz	45043452	Drechow	45363148	Olganitz
44883228	Schlamau	45043468	Velgast	45363164	Triestewitz
44883276	Gräningen	45043484	Sundische Wiese	45363196	Neuerstadt
44883292	Kleßen-Görne-West	45202748	Siegsdorf	45363212	Jüterbog-Südost
44883308	Bückwitz	45202764	Holzhausen / Dieperting	45363228	Luckenwalde-Nordwest
44883324	Wulkow	45202780	Kirchweidach-Nordost	45363244	Schiaß
44883340	Wittstock-Süd	45202796	Neuötting	45363292	Velten
44883356	Wredenhagen	45202812	Huldsessen	45363308	Teschendorf
44883372	Lexow	45202828	Ruhstorf	45363324	Kraatz
44883388	Neu Gaarz	45202844	Landau an der Isar	45363372	Wanzka
44883404	Hohen Demzin	45202860	Straßkirchen	45363404	Altentreptow
44883436	Repnitz	45202876	Gaishausen	45363420	Alt Tellin
44883452	Marlow	45202892	Kasparzell	45363436	Görmin
44883468	Hessenburg	45202908	Kothmaißling	45363452	Klein Petershagen
44883484	Prerow	45203052	Schwarzenberg-Nordost	45363468	Mellnitz
45042764	Roitham	45203068	Brünlos	45363484	Dreschwitz
45042780	Peterskirchen-Nordwest	45203084	Chemnitz-Rabenstein	45363500	Vieregge
45042812	Egglkofen-Ost	45203100	Wiederau	45522812	Kößlarn

45042828	Loizenkirchen	45203116	Colditz-Ost	45522828	Haarbacherloh
45042844	Moosthenning	45203132	Gastewitz	45522844	Hofkirchen / Pleinting
45042860	Pönning	45203148	Lossatal-Voigtshain	45522860	Grattersdorf
45042876	Oberzeitldorn	45203164	Großwig	45523068	Sorgau
45042892	Völling	45203180	Axien	45523084	Kleinhartmannsdorf
45042908	Hitzelsberg	45203196	Gentha	45523100	Großschirma
45042924	Heinrichskirchen	45203212	Eckmannsdorf	45523116	Raußlitz
45043068	Reinsdorf-Ost	45203228	Niebel	45523132	Prausitz
45043084	Ebersbach	45203276	Wustermark	45523164	Zinsdorf
45043100	Neuenmörbitz	45203308	Pabsthum	45523180	Jagsal
45043116	Bad Lausick-Südwest	45203356	Wesenberg	45523196	Knippelsdorf
45043132	Klinga	45203388	Groß Flotow	45523212	Petkus-Süd
45043148	Groitzsch	45203404	Tüzen	45523244	Glienick
45043196	Pratau	45203420	Glendelin	45523308	Kreuzbruch
45043212	Boßdorf	45203452	Grimmen-Nord	45523340	Templin-West
45043228	Mörz	45203468	Stralsund-Süd	45523372	Krumbeck
45043260	Jeserig	45362748	Anger	45523388	Kublank
45043292	Jahnberge	45362796	Stammham	45523404	Salow
45043308	Protzen	45362812	Fraunleiten	45523420	Medow
45043340	Zechlin-West	45362844	Eschlbach	45523436	Züssow
45043356	Lärz	45362860	Deggendorf-Natternberg	45523452	Gahlkow
45043388	Torgelow am See	45363052	Königswalde	45523500	Neddesitz
45682812	Reindlöd	45843196	Schlabendorf	46003372	Woddow
45682828	Aspertsham	45843212	Lubolz	46003388	Rothenklempenow
45682844	Ranzing	45843244	Görsdorf	46163100	Sebnitz-Mittelndorf
45683084	Burkersdorf	45843308	Eberswalde-Tornow	46163116	Oberottendorf
45683116	Röhrsdorf	45843324	Ziethen	46163132	Siebitz
45683132	Lenz	45843340	Greiffenberg-Nordwest	46163148	Wittichenau-Saalau
45683180	Kirchhainer Wiesen	45843356	Bertikow	46163228	Friedland-Günthersdorf
45683196	Wüstermarke	45843372	Göritz-Tornow	46163260	Petersdorf
45683212	Golßen	45843388	Belling	46163276	Diedersdorf
45683244	Bestensee	46002844	Thalberg	46163292	Letschin
45683292	Bernau-Ost	46003100	Pirna-Südost	46323228	Treppeln
45683388	Schwarzensee	46003132	Oberlichtenau	46323276	Reitwein
45683404	Lübkowsee	46003196	Göritz	46483116	Niedercunnersdorf
45683420	Bargischow	46003260	Fürstenwalde-Nordost	46483132	Weißenberg-Nordost
45683436	Zemitz	46003276	Müncheberg-Nordwest	46483148	Kreba
45842844	Oberkümmering	46003292	Möglin	46483164	Weißkeißel
45843084	Falkenhain	46003308	Neuranft	46643116	Kiesdorf auf dem Eigen
45843100	Kreischa	46003340	Heinersdorf / Landin	46643132	Kunnersdorf
45843148	Ortrand-Ost	46003356	Wartin		

Step 6: Specify locations of wild bee surveys within each landscape quadrat

The algorithm for the initial determination nesting aid locations, as implemented in R, version 4.0.2 [71], can be found as supplementary file LUCAS_NestingAids.R together with the SHP files of LUCAS grid points and landscape quadrats, and the CSV file with LUCAS ids. Please note that the Basic DLM data used to distinguish land use types are administrative data available for purchase via https://gdz.bkg.bund.de/index.php/default/digitales-basis-landschaftsmodell-kompakt-basis-dlm-kompakt.html (accessed on 14 July 2022).
